# Possible correlation between gut microbiota and immunity among healthy middle-aged and elderly people in southwest China

**DOI:** 10.1186/s13099-018-0231-3

**Published:** 2018-02-09

**Authors:** Xi Shen, Junjie Miao, Qun Wan, Shuyue Wang, Ming Li, Fangfang Pu, Guoqing Wang, Wei Qian, Qian Yu, Francesco Marotta, Fang He

**Affiliations:** 1 Department of Nutrition, Food Safety and Toxicology, West China School of Public Health, Sichuan University, No. 17 People’s South Road, Chengdu, 610041 Sichuan People’s Republic of China; 2 School of Public Health, Hebei Medical University, Shijiazhuang, 050017 Hebei People’s Republic of China; 3 Department of Nutrition, Hangzhou Children’s Hospital, Hangzhou, 310006 Zhejiang People’s Republic of China; 4 Department of Public Health Laboratory Sciences, West China School of Public Health, Sichuan University, Chengdu, 610041 Sichuan People’s Republic of China; 5 By-health Co. Ltd., No. 3 Kehui 3rd Street, No.99 Kexue Avenue Central, Huangpu District, Guangzhou, 510663 People’s Republic of China; 6 ReGenera Research Group for and Gender Healthy Aging Unit, Montenapoleone Medical Center, Aging Intervention Corso Matteotti, 1/A, 20121 Milan, Italy

**Keywords:** Ageing, Gut microbiome, Immunosenescence, Gene sequencing

## Abstract

**Background:**

The present study was conducted to investigate the possible association between gut microbes and immunity among healthy middle-aged and elderly individuals in southwest China. A total of 148 healthy adults aged ≥ 50 years were divided into two age groups: middle-aged group (50–59 years; n = 67, 54.13 ± 3.32) and elderly group (≥ 60 years; n = 81, 64.70 ± 3.93). Blood samples were collected to measure serum immune and biochemical indices. Gut microbiota compositions of the groups were characterized on the basis of faecal DNA using 16S rRNA gene sequencing.

**Results:**

Among the detected gut microbes, the presence of *Alistipes* was negatively correlated with age in both groups. In the middle-aged group, age was negatively correlated with the presence of *Desulfovibrio* and *Faecalibacterium*. In the elderly group, *Coprococcus* was present at significantly higher levels; age was negatively correlated with the presence of *Lachnobacterium*, *Oxalobacter* and the Chao index, whereas positively correlated with the presence of *Sutterella.* In the middle-aged group, the presence of *Bacteroidetes* was positively correlated with serum immunoglobulin G (IgG) levels and the percent of CD8^+^ T cells and negatively correlated with the CD4^+^/CD8^+^ ratio; the presence of *Firmicutes* was negatively correlated with IgM levels; *Bacteroidetes/Firmicutes* ratio was positively correlated with IgG and IgM levels and Simpson index was negatively correlated with the percent of CD8^+^ T cells and positively correlated with CD4^+^/CD8^+^ ratio. In the elderly group, the presence of *Verrucomicrobia* (identified as genus *Akkermansia*) was positively correlated with IgA levels and the percent of CD8^+^ T cells and negatively correlated with the percent of CD4^+^ T cells and CD4^+^/CD8^+^ ratio; the Chao index and observed species were positively correlated with IgA levels.

**Conclusions:**

These results indicated that ageing could significantly correlate with the composition of gut microbiota in terms of quantity and quality. Changes in gut microbiota caused by ageing, characterized by decreased *Bacteroidetes* levels, might be associated with immunosenescence among healthy middle-aged and elderly people in southwest China.

**Electronic supplementary material:**

The online version of this article (10.1186/s13099-018-0231-3) contains supplementary material, which is available to authorized users.

## Background

With the development of medical and sanitary conditions over the past 50 years, the average human life expectancy in most countries has significantly increased [1]. In China, owing to the long life expectancy and low birth rate, the fraction of the population aged > 60 years will outnumber children aged < 5 years by 2020, and between 2015 and 2050, Chinese population will rapidly double from 12 to 22% of the world’s population, reaching approximately 2 billion by 2050. The rate of ageing in Chinese population is fast, and the proportion of elderly people will reach 28% of the population by 2040 [2].

Ageing is always accompanied by poor health and declining physiological functions, elevating incidences of infectious diseases and a number of pathologies that have a common inflammatory origin, such as atherosclerosis and cardiovascular disease, type II diabetes, arthritis, dementia, Alzheimer’s disease, osteoporosis or cancer [3]. This poses great challenges and burdens pertaining to social health and well-being, healthcare and economic growth; therefore, attention to healthy ageing prospects is crucial.

Immunosenescence is a likely mechanism underlying these ageing-related diseases. It is defined as the state of deregulated immune function contributing to increased susceptibility of the elderly to infection and possibly to autoimmune diseases and cancer [4]. When immunosenescence occurs, the functional capacity of the immune system of the host gradually declines with age. The most marked changes in the immune system that occur with age involve the T cell compartment, the arm of the immune system that protects against pathogens and tumours [4, 5]. Therefore, immunosenescence can reflect ageing-related decrease in immune function at the cellular and serological levels [6] as well as can concomitantly occur with the hyper stimulation of both innate and adaptive immune systems that may result in a low-grade chronic state of inflammation known as inflammaging [7, 8]. One of the key underlying mechanisms of this phenomenon is thymus involution, which is almost complete by the age of 60 years.

The gut microbiota, including a wide variety of microorganisms in large numbers inhabiting in the gastrointestinal (GI) tract, plays an important role in maintaining GI homeostasis and regulating host metabolism as an integral part of the host system. Studies have shown that each individual has at least 160 taxonomic species belonging to 1000–1150 prevalent bacterial species; their collective genome (‘microbial group’) comprises at least 100 times as many genes as the human genome [9]. The composition of the microbiota develops during the first few years of life and is relatively stable during adult life [9, 10]. Gut microbiota could be influenced by host genetics, health, diet, ageing and probiotics [10–13]. Ageing-related changes in gut microbial composition may be associated with many diseases and disorders in the elderly [11, 14, 15]. Some recent studies have also indicated that gut microbes, particularly certain selected strains, might enhance cell-mediated immunity in host animals, thereby altering ageing-related immunosenescence [16]. However, mechanisms and timing of changes in gut microbial composition with age remain unclear, and only a few previous studies have focussed on the potential association between human immunosenescence and gut microbiota during the ageing process.

The present study was conducted to characterize ageing-related changes in properties of gut microbiota from adult to elderly stages and to investigate whether these changes are associated with host immunity.

## Methods

### Study design, subjects and sample collection

A total of 148 healthy adults aged ≥ 50 years were selected and divided into two groups on the basis of age. Middle-aged group included 67 individuals aged 50–59 years, and elderly group included 81 individuals aged ≥ 60 years. Most of the participants had good physical and mental health and were from the labour union of West China School of Public Health of Sichuan University, Chengdu, China. All participants provided written informed consents to participate in the study. Individuals who were hospitalized in the past 3 months; had histories of heart failure, myocardial infarction, severe kidney and cerebrovascular diseases, or diabetes; used medications for these diseases or had participated in other clinical trials were excluded.

All participants underwent basic medical examinations and responded to questionnaires regarding their diet and lifestyle. Blood samples were collected to measure serum immune and biochemical indices. Faecal samples were collected and stored at − 80 °C until DNA extraction.

Immune functions were assessed on the basis of lymphocyte subsets detected using flow cytometry (BD FACSCalibur, BD, Inc., USA), including total T cells (CD3^+^), helper T cells (CD4^+^) and cytotoxic T cells (CD8^+^) (CD4-FITC/CD8-PE/CD3-PerCP, BD, Inc., USA), and immunoglobulin (Ig)A, IgG and IgM levels detected using an automatic specific protein analyser (IMMAGE 800, Beckman Coulter, Inc., USA). Nutritional status was reflected by glucose, total cholesterol (TC), triglycerides (TG), high-density lipoproteins (HDL), low-density lipoproteins (LDL), Uric acid and Blood urea nitrogen detected using automatic biochemical analyser [17]. All analyses were conducted using standard procedures approved by the West China Medical Detection Institute, Sichuan University (Chengdu, China). All procedures in the study were conducted in accordance with the Helsinki declaration and were reviewed and approved by the Ethics Committee of West China School of Public Health (No. 4 West China Teaching Hospital) of Sichuan University.

### DNA extraction and 16S rRNA sequencing

DNA was extracted from faecal samples using acommercial DNA isolation kit (TIANamp Stool DNA Kit, TIANGEN, Beijing, China) following the manufacturer’s protocol with some modifications [18]. DNA concentration and purity were analysed using Nanodrop 2000 ultraviolet–visible microspectrophotometer (Thermo-Fisher scientific, Inc., USA). The V4 region of 16S rRNA was amplified using 515F and 806R fusion primers containing Illumina adapters and sample-specific barcodes. PCR amplicons were purified using magnetic beads. Library clusters were paired-end sequenced (2 × 250 bp) on an Illumina Miseq Benchtop Sequencer at Beijing Genomics Institute (Beijing, China).

Low-quality reads were filtered; paired-end reads were assembled using FLASH (v1.2.11), and chimeric sequences were removed using the UCHIME (v4.2.40) software aligned to the gold database (v20110519). The USEARCH (v7.0.1090) software was used to identify representative sequences for each operational taxonomic unit (OTU), generated from complete linkage clustering with a 97% similarity against de novo clustered sequences and aligned to the Greengenes database (v201305 [8]). OTU tables with percent relative abundances were then annotated at taxonomic levels from phylum to species.

### Statistical analysis

The data were presented as means ± standard deviations. The differences between groups were analysed by t-test or Wilcoxon rank-sum test. Spearman’s rank correlation coefficient was used for assessing ageing-related changes in microbial compositions and correlation between bacterial relative abundance and immunity indices. Differences with *P* < 0.05 were considered to be statistically significant.

## Results

### Subjects

Characteristics of the participants selected for this study are described in Table 1. The mean age of the participants in the middle-aged group was 54.13 ± 3.32 years; this group comprised 27 men and 40 women. Furthermore, the mean age of the participants in the elderly group was 64.70 ± 3.93 years; this group comprised 41 men and 40 women (with only three individuals aged > 70 years).

**Table 1:** Characteristics of the study participants

	Middle-aged group (50–59 years)	Elderly group (≥ 60 years)	*P* value
Age	54.13 ± 3.32	64.70 ± 3.93	0.000
n	67	81	–
Gender (male/female)	27/40	41/40	–
BMI (kg/m^2^)	23.41 ± 2.79	23.36 ± 2.75	0.906
IgA (g/L)	2.38 ± 0.99	2.59 ± 0.96	0.198
IgG (g/L)	13.97 ± 2.38	14.03 ± 2.20	0.873
IgM (g/L)	1.25 ± 0.45	1.16 ± 0.49	0.271

Values are presented as mean ± SD or frequencies, tested for differences between the groups using t test

### Physiological and serum immune functions of participants

No differences were observed in the baseline characteristics, except age, between the middle-aged and elderly groups. All serum indices were normal (Additional file 1: Table S1).

### Faecal microbe composition

**Fig. 1:**
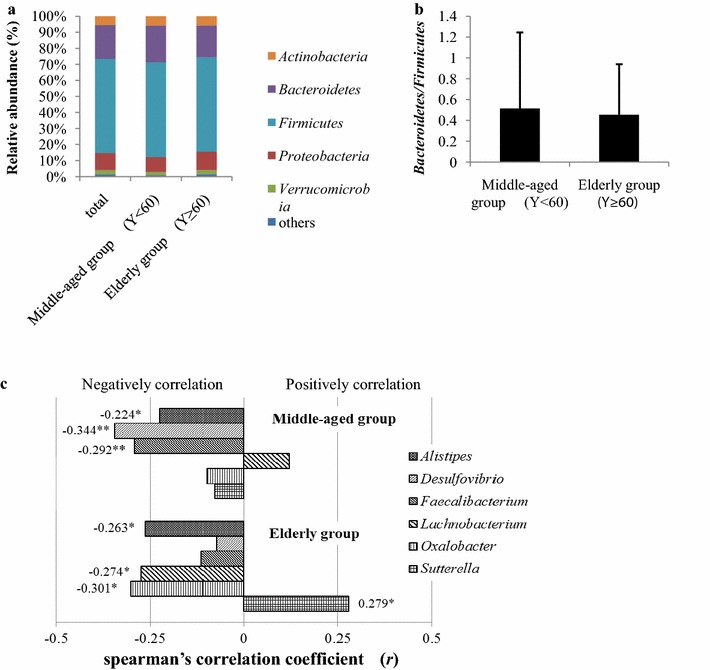
Composition of gut microbiota. **a** Composition of gut microbiota at the phylum level. Phyla with relative abundance greater than 0.5% are presented. The lower abundant phyla are grouped as ‘Others’. *Bacteroidetes* level was lower in the elderly group than that in the middle-aged group, but no significant differences were observed. **b** *Bacteroidetes*/*Firmicutes* ratio. Middle-aged group, 0.51; elderly group, 0.46. *Bacteroidetes*/*Firmicutes*ratio was lower in the elderly group than that in the middle-aged group, but no significant differences were observed. **c** Correlation between age and gut microbial composition at genus level. Spearman’s correlation coefficient was calculated between microbial composition and age. * and ** indicate significant difference at a *P* value of < 0.05 and < 0.01, respectively

A total of 7,788,618 high-quality reads (with 57,744 reads eliminated during quality control) were obtained from the 148 samples, with 52,625 ± 22,913 reads per sample, which were clustered into 1560 OTUs with 97% similarity. Taxonomy-based analysis revealed that the faecal microbiota of all participants most abundantly comprised species belonging to the phyla *Firmicutes* (58.80%) and *Bacteroidetes* (21.06%), followed by those belonging to *Proteobacteria* (10.56%), *Actinobacteria* (5.51%) and *Verrucomicrobia* (2.70%). The distributions of the major phyla between the two age groups were similar to the results of the total, *Bacteroidetes* level was lower in the elderly group (19.80%) than that in the middle-aged group (22.96%), but no significant differences were observed between the two groups (Fig. 1a). As shown, *Bacteroidetes*/*Firmicutes*ratio was lower in the elderly group than that in the middle-aged group, but no significant differences were observed between the two groups (Fig. 1b).

At the genus level, only the presence of species belonging to the genus *Coprococcus* (*P* = 0.003) was significantly higher in the elderly group than that in the middle-aged group (Table 2 and Additional file 1: Figure S1). No other differences in microbial composition were observed between the two groups. However, obvious correlations were observed between age and the composition of gut microbiota in both the age groups. The presence of *Alistipes* was negatively correlated with age in both the groups (middle-aged group, *r* = − 0.224, *P* = 0.047; elderly group, *r* = − 0.263, *P* = 0.029). Furthermore, in the middle-aged group, age was negatively correlated with the presence of *Desulfovibrio* (*r* = − 0.344, *P* = 0.002) and *Faecalibacterium* (*r* = − 0.292, *P* = 0.009). In the elderly group, age was negatively correlated with the presence of species belonging to the genus *Lachnobacterium* (*r* = − 0.274, *P* = 0.023) and *Oxalobacter* (*r* = − 0.301, *P* = 0.012) and positively correlated with those belonging to the genus *Sutterella* (*r* = 0.279, *P* = 0.020) (Fig. 1c).

**Table 2:** Comparison of gut microbiota at genus level

Genus	Middle-aged group (%)	Elderly group (%)	*P* value
*Akkermansia*	2.02	3.49	0.099
*Bacteroides*	16.96	12.89	0.490
*Bifidobacterium*	3.94	6.20	0.332
*Blautia*	3.08	2.76	0.795
*Clostridium*	2.69	2.64	0.639
*Coprococcus*	1.51	2.25	0.003*
*Desulfovibrio*	0.45	0.28	0.181
*Dialister*	0.65	1.73	0.538
*Dorea*	0.93	1.10	0.775
*Escherichia*	6.65	7.59	0.464
*Eubacterium*	0.80	0.40	0.835
*Faecalibacterium*	16.56	12.99	0.109
*Haemophilus*	0.28	0.90	0.114
*Lachnospira*	0.37	0.58	0.058
*Lactobacillus*	0.51	0.17	0.500
*Megamonas*	4.06	0.90	0.720
*Oscillospira*	0.83	0.99	0.867
*Parabacteroides*	0.51	1.05	0.780
*Phascolarctobacterium*	1.88	0.83	0.436
*Prevotella*	2.94	3.17	0.371
*Roseburia*	4.02	5.08	0.279
*Ruminococcus*	5.15	5.89	0.708
*SMB53*	1.08	0.66	0.742
*Streptococcus*	2.02	3.14	0.226
*Sutterella*	1.06	0.72	0.840
*Veillonella*	0.43	1.19	0.095
Unclassified	16.04	18.24	0.608
Others (< 0.5%)	2.58	2.20	0.901

Genera with the relative abundance of more than 0.5% are presented as mean and compared between the middle-aged and elderly groups using Wilcoxon rank-sum test. The lower abundant genera are grouped as ‘Others’. * indicates significant difference at a *P* value of < 0.05. At the genus level, only the presence of species belonging to the genus *Coprococcus* was significantly higher in the elderly group

### Richness and diversity of faecal microbiota

**Fig. 2:**
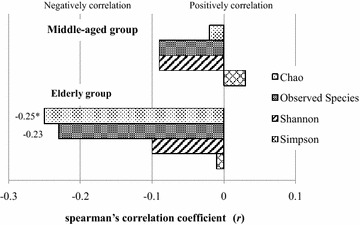
Correlation between age and the richness and diversity of gut microbiota. Spearman’s correlation coefficient was calculated between age and richness and diversity indexes of gut microbiota. * indicates significant difference at a *P* value of < 0.05

In the elderly group, age was negatively correlated with the Chao index (*r* = − 0.250, *P* = 0.038) and was negatively correlated with the observed species (*r* = − 0.231, *P* = 0.057); however, no significant correlations were observed in the middle-aged group (Fig. 2).

### Correlation analysis between gut microbiota and immune indices

The possible association between gut microbiota and immunoglobulin levels, including IgA, IgG and IgM, was analysed (Table 3). In terms of taxonomy, in the middle-aged group, the presence of species belonging to the phylum *Bacteroidetes* was positively correlated or tended to be positively correlated with IgG (*r* = 0.246, *P* = 0.029) or IgM (*r* = 0.218, *P* = 0.054) levels; those belonging to the phylum *Firmicutes* were negatively correlated with IgM levels (*r* = − 0.243, *P* = 0.031) and the *Bacteroidetes/Firmicutes* ratio was positively correlated with serum IgG (*r* = 0.222, *P* = 0.049) and IgM (*r* = 0.243, *P* = 0.031) levels. In the elderly group, the presence of species belonging to the phylum *Verrucomicrobia* (identified as genus *Akkermansia*, *r* = 0.428, *P* < 0.001) was positively correlated with serum IgA levels. With respect to richness and diversity, the Chao index (*r* = 0.261, *P* = 0.030) and the presence of observed species (*r* = 0.280, *P* = 0.020) were positively correlated with serum IgA levels in the elderly group. In addition, no significant correlations were found between age and serum IgA, IgG or IgM levels.

**Table 3:** Correlation of gut microbiota and immune indices: correlation between gut microbiota and serum immunoglobulin levels

	Middle-aged group			Elderly group		
	IgA	IgG	IgM	IgA	IgG	IgM
Phylum						
*Actinobacteria*	0.023	− 0.012	− 0.069	0.001	0.157	0.137
*Bacteroidetes*	0.158	0.246*	0.218	− 0.125	− 0.102	− 0.062
*Firmicutes*	− 0.009	0.085	− 0.243*	0.102	0.041	0.086
*Proteobacteria*	− 0.021	− 0.208	0.154	0.004	− 0.119	0.131
*Verrucomicrobia*	− 0.049	− 0.011	0.001	0.428**	− 0.040	0.025
*Bacteroidetes*/*Firmicutes*	0.136	0.222*	0.243*	− 0.107	− 0.099	− 0.074
Richness/diversity						
Chao	0.050	0.063	− 0.087	0.261*	0.094	− 0.145
Observed Species	0.092	0.083	− 0.065	0.280*	0.048	− 0.227
Shannon	0.083	0.093	0.020	0.199	− 0.043	− 0.020
Simpson	− 0.079	− 0.082	− 0.022	− 0.139	0.094	− 0.076

Spearman’s correlation coefficient was calculated between the presence of gut microbiota and serum immunoglobulin levels. * and ** indicate significant difference with a *P* value of < 0.05 and < 0.01, respectively. Observed species value and chao value can reflect the species richness of community. Shannon value and simpson value can reflect the species diversity of the community

Further, the correlation between gut microbiota and T cell levels was analysed (Table 4). In the middle-aged group, the presence of species belonging to the phylum *Bacteroidetes* was positively correlated with the percent of CD8^+^ T cells (*r* = 0.254, *P* = 0.041) and negatively correlated with CD4^+^/CD8^+^ ratio (*r* = − 0.244, *P* = 0.049). The Simpson index was negatively correlated with the percent of CD8^+^ T cells (*r* = − 0.248, *P* = 0.046) and positively correlated with CD4^+^/CD8^+^ ratio (*r* = 0.260, *P* = 0.036). In the elderly group, the presence of species belonging to the phylum *Verrucomicrobia* (*r* = 0.197, *P* = 0.041) was positively correlated with the percent of CD8^+^ T cells but negatively correlated with the percent of CD4^+^ T cells (*r* = − 0.288, *P* = 0.02), and CD4^+^/CD8^+^ ratio (*r* = − 0.299, *P* = 0.021, respectively). Similarly, no significant correlations were observed between age and T cell levels.

**Table 4:** Correlation of gut microbiota and immune indices: correlation of gut microbiota and T cell levels

	Middle-aged group				Elderly group			
	CD3 +	CD4+ %	CD8+ %	CD4+/CD8+	CD3+	CD4+ %	CD8+ %	CD4+/CD8+
Phylum								
*Actinobacteria*	0.149	0.098	− 0.186	0.126	0.000	0.010	0.039	− 0.014
*Bacteroidetes*	− 0.175	− 0.200	0.254*	− 0.244*	− 0.058	− 0.107	0.135	− 0.120
*Firmicutes*	0.124	− 0.239	0.156	− 0.186	0.137	− 0.045	0.005	− 0.022
*Proteobacteria*	0.158	− 0.016	− 0.052	0.032	− 0.059	0.088	0.000	0.039
*Verrucomicrobia*	− 0.052	0.173	− 0.174	0.179	0.086	− 0.288*	0.197*	− 0.299*
*Bacteroidetes*/*Firmicutes*	− 0.215	− 0.171	0.220	− 0.205	0.026	0.113	− 0.004	0.053
Richness/diversity								
Chao	− 0.145	− 0.084	0.122	− 0.130	0.176	− 0.203	0.128	− 0.166
Observed Species	− 0.206	− 0.125	0.163	− 0.169	0.212	− 0.186	0.121	− 0.153
Shannon	− 0.159	− 0.194	0.230	− 0.236	0.087	− 0.092	− 0.007	− 0.031
Simpson	0.138	0.221	− 0.248*	0.260*	− 0.032	0.044	0.054	− 0.018

The Spearman’s correlation coefficient was calculated between gut microbiota and T cell levels. * and ** significant difference at a *P* value of < 0.05 and < 0.01, respectively

## Discussion

Several studies have observed differences in gut microbial composition between the elderly and younger people. Such ageing causes changes in gut microbial community and may therefore influence host physiological functions, including immunity, and could be involved with potent pathogens of various ageing-related diseases and disorders, particularly of noncommunicable diseases [19]. For example, immunosenescence could be caused by an abnormally activated immune response to gut microbiota, which might be due to diminished mucosal tolerance, ageing-related changes in gut microbiota or both [20]. Evidence has been obtained from studies in which the administration of dietary probiotics improved systemic immune responsiveness [21]. Therefore, immunosenescence and changes in gut microbiota might be associated with each other and might concurrently affect health. However, the association between these two changes has not been studied in great depth. In the present study, the associations between age, gut microbiota composition and immunity in both adults and the elderly were analysed with participants from southwest China, are relatively isolated region with its own characterized local culture and diet. They represent the general population of a restricted geographic area and can be considered as a relatively homogeneous cohort in terms of their lifestyle and dietary habits. In China, most people retire at the age of 60 years, with their lifestyle greatly changing after this age. Based on this, the study participants were divided into two groups as follows: middle-aged group (50–59 years) and elderly group (≥ 60 years).

Human gut microbiota comprises approximately 500–1000 species that belong to only a few known bacterial phyla. The most abundant phyla are *Firmicutes*, *Bacteriodetes*, *Proteobacteria*, *Verrumicrobia* and *Actinobacteria* [22]. The *Bacteroidetes*/*Firmicutes* ratio varies from 2.5 to 0.09 between infants and adults, with 1.6 in the elderly [23]. Low *Bacteroidetes/Firmicutes* ratio has been suggested as one of the hallmarks of human gut microbiota because this ratio has been observed to be lower in obese individuals in some studies in humans [24]. In the present study, *Firmicutes* (58.80%) and *Bacteroidetes* (21.06%) were the most abundant, followed by *Proteobacteria* (10.56%), *Actinobacteria* (5.51%) and *Verrucomicrobia* (2.70%), which were detected in all of the tested participants. These results are in agreement with those reported by the previous studies [22]. However, *Bacteroidetes/Firmicutes* ratio was almost 0.35, which was relatively lower than those reported in other elderly people [23]. Overall, the results obtained in the present study indicate that although all the tested participants are clinically healthy, they are at a potential risk of metabolic disorders in the context of their intestinal microbial composition. Conversely, these results might raise a question regarding the reported association between low *Bacteroidetes/Firmicutes* ratios and metabolic disorders considering that the tested participants are from the region in southwest China with a less obese and overweight population, whose diets are well known for being healthy.

The present study also indicated that some genus compositions, including *Alistipes*, *Desulfovibrio*, *Faecalibacterium*, *Lachnobacterium* and *Oxalobacter*, markedly decreased with age in the adults aged ≥ 50 years, whereas the percent of genus *Sutterella* significantly increased in the elderly group. Earlier studies have demonstrated a significant decrease in beneficial bacteria, such as *Bacteroides*, *Lactobacillus* and *Bifidobacterium*, with age [15]. Elderly individuals had a higher proportion of species belonging to phylum *Bacteroidetes*, suggesting that *Bacteroidetes* are beneficial to the elderly [3]; However, in the present study, *Alistipes* (in the phylum *Bacteroidetes*) decreased with age, both in middle-aged and elderly groups. *Faecalibacterium* and *Lachnobacterium* from the phylum *Firmicutes* decreased with age in middle-aged and elderly groups, respectively. *Faecalibacterium*, particularly *F. prausnitzii* is one of the important commensal microbes of human gut microbiota. They can synthesize butyrate and other short-chain fatty acids through the fermentation of dietary fibre to promote host health. In healthy adults, *F. prausnitzii* represents more than 5% of the intestinal bacteria. Lower than normal levels of *F. prausnitzii* are have been known to be associated with the development of Crohn’s Disease, obesity, asthma and major depressive disorder [25–27]. *Lachnobacterium* belongs to the family *Lachnospiraceae*; members of this family may provide protection against colon cancer in humans by producing butyric acid [28]. In addition, *Desulfovibrio* and *Oxalobacter* (in the phylum *Proteobacteria*) decreased in the middle-aged and elderly groups, respectively. *O. formigenes*, a member of the genus *Oxalobacter*, is an obligate anaerobe and digests oxalate as a carbon and energy source for cells. A lack of *O. formigenes* colonizing the colon is a risk factor for the development of hyperoxaluria and calcium oxalate stones. Further, levels of the genus *Coprococcus* were significantly higher in the elderly group than those in the middle-aged group. *Coprococcus*is a genus of anaerobic cocci; compared with younger adults, *Enterococci*levels are higher in the elderly, as reported in previous Asian studies [19]. Based on these previous studies, the ageing-related decreases in *Alistipes*, *Desulfovibrio*, *Faecalibacterium*, *Lachnobacterium* and *Oxalobacter* among the tested participants could negatively affect their health. To the best of our knowledge, this is the first study to demonstrate that levels of *Alistipes*, *Desulfovibrio*, *Faecalibacterium*, *Lachnobacterium, Oxalobacter* and *Coprococcus* could be decreased during the process of ageing although the tested subjects were specific in terms of their geographic origin and life style. Our findings indicate that ageing could negatively affect the quality and quantity of human gut microbiota though different mechanisms across different populations and that its effects are not limited to *Bifidobacteria* and *Lactobacillus.*

In addition to gut microbial composition, several studies have shown lower richness and diversity of gut microbiota colonized in nonagenarians or centenarians than those in young adults [29]. In this study, the microbial richness (Chao index and observed species) of the tested participants were observed to decrease with age in the elderly group. These results were consistent with those reported in the previous studies although ageing-related changes in gut microbiota have been observed to occur at a much earlier age. Recent studies have suggested that the loss of richness and diversity in gut microbiota, or called dysbiosis is associated with increased frailty, which might be caused by the accumulation of disorders and an imbalance in the intestinal ecosystem that leads to decreased immunity and increased susceptibility to pathogens [30].

Immunosenescence is most likely a combination of autoimmunity, immunodeficiency and immune dysregulation, which accompanies a chronic state of low-grade inflammation associated with physiological ageing [31]. Evidence has been presented to support the hypothesis that the inflammatory status of the elderly might be partly caused or aggravated by changes in microbial proportions [3], such as high plasma proinflammatory cytokine (IL-6 and IL-8) levels, which are associated with the presence of bacteria belonging to the phylum *Proteobacteria* [32].

In the middle-aged group, a significantly positive correlation was observed between the presence of phylum *Bacteroidetes* and IgG levels, which were positively correlated with IgM levels; whereas the presence of *Firmicutes* was negatively correlated with IgM levels. *Bacteroidetes/Firmicutes* ratio was also positively correlated with serum IgG and IgM levels. Antibodies, such as IgG and IgM, as the key components of host humoral immunity can play an important role in protecting the host from various infections. Our findings indicated that ageing-related decrease in *Bacteroidetes* levels and *Bacteroidetes/Firmicutes* ratio could be associated with the dysregulation of host humoral immunity.

In terms of cellular immunity, the presence of phylum *Bacteroidetes* was positively correlated with the percent of CD8^+^ T cells and negatively correlated with CD4^+^/CD8^+^ ratio. CD8^+^ T cells are also known as cytotoxic T cells because they express the CD8 glycoprotein on their surfaces, destroy virus-infected cells and tumour cells and are also implicated in transplant rejection. Our findings indicated that *Bacteroidetes* might stimulate host cellular and humoral immunity. Therefore, these results indicate that ageing-related decrease in *Bacteroidetes* observed in the present study could be associated with the dysregulation of host cellular immunity.

In the elderly group, the presence of phylum *Verrucomicrobia* was positively correlated with serum IgA levels and the percent of CD8^+^ T cells but negatively correlated with the percent of CD4^+^ T cells and CD4^+^ to CD8^+^ ratio. *A. muciniphila* is the only species of *Verrucomicrobia* that shows anti-inflammatory effects in humans, and studies have shown that the presence of this species is associated with the development of irritable bowel syndrome, obesity and type-2 diabetes [33–36]. Therefore, our findings indicate that the microbes taxonomically belonging to *Verrucomicrobia* may be a group of microbes that benefit the host. Therefore, *Verrucomicrobia* could be considered as an important biomarker to monitor host immune status in the elderly.

In the present study, the percent of CD8^+^ T cells decreased with decrease in the diversity (Simpson index) of gut microbiota in the middle-aged group, whereas decrease in diversity was not significant in the elderly group. In contrast, serum IgA levels decreased with decrease in the richness (Chao index and observed species) of gut microbiota in the elderly group. Therefore, our findings indicate that ageing-related decrease in gut microbial diversity could be associated with the dysregulation of host immunity with various faces dependent on the different ageing process. These results demonstrate that gut microbiota could be closely associated with the immune status of the elderly and that they could act as potent pathogens to cause or influence various ageing-associated diseases and disorders.

## Conclusion

The results obtained in the present study indicate a potential specific symbiotic relationship between human gut microbiota and host immunity. Further, each of gut microbes could affect the development and function host immunity through their own characteristic manners. Ageing-related changes in gut microbiota could be associated with abnormal immunity, such as immunosenescence. Therefore, homeostasis is in gut microbes would be crucial to maintain the proper function of immunity system, particularly in the elderly. However, further studies focussing on diet, lifestyle, living environment and other factors across different populations are required to confirm such a possibility.

## Additional file

**Additional file 1: Table S1.** Serum indices and normal range. **Figure S1.** Composition of gut microbiota at the genus level.

## Abbreviations

Ig: immunoglobulin
GI: gastrointestinal
TC: total cholesterol
TG: triglycerides
HDL: high-density lipoproteins
LDL: low-density lipoproteins
OTU: operational taxonomic unit
