# Supplementary material for: Possible correlation between gut microbiota and immunity among healthy middle-aged and elderly people in southwest China
Source: Gut Pathog. 2018 Feb 9;10:4. doi: 10.1186/s13099-018-0231-3 (PMC5806246; doi:10.1186/s13099-018-0231-3)
Supplement: Supplementary file 1 — Additional file 1: Table S1. Serum indices and normal range. Figure S1. Composition of gut microbiota at the genus level. [file 13099_2018_231_MOESM1_ESM.pdf]

Additional file 1

Table S1. Serum indices and normal range

Indicators	Middle-aged group	Elderly group	Normal range
Age(year)	54.13±3.32	64.70±3.93	---
GLU(mmol/L)	6.19±1.45	5.89±0.90	<6.1mmol/L
HbA1c(%)	5.97±1.10	5.86±0.86	<7.0%
SSP（mmHg）	126.59±17.13	124.72±14.88	≤140mmHg
DBP（mmHg）	76.11±11.14	75.88±10.32	≤90mmHg
TC（mmol/L）	5.45±0.95	5.36±0.91	2.9-6.0mmol/L
TG（mmol/L）	1.43±0.80	1.57±1.07	0.39-1.76mmol/L
HDL（mmol/L）	1.47±0.53	1.40±0.42	1.29-1.55 mmol/L
LDL（mmol/L）	2.99±0.86	3.23±0.99	<3.37mmol/L
UA （μmol/L）	364.68±84.56	371.17±90.57	90-420umol/L
BUN （mmol/L）	5.54±1.37	5.49±1.44	3.2-7.1mmol/L
SCR（μ mol/L）	101.58±25.94	113.16±30.63	44-133 μmol/L
IgA（ mg /mL）	2.38±0.99	2.59±0.97	76 -3.90 mg /mL
IgG（ mg /mL）	13.97±2.38	14.03±2.19	6 -16 mg /mL
IgM（ mg /mL）	1.25±0.45	1.16±0.49	0.40-3.45 mg /mL
CD3+ T cell counts	1257.50±453.29	1296.12±395.65	≥615
CD4+ T cell %（ %）	55.06±11.00	53.47±10.96	——
CD8+ T cell %（ %）	40.62±10.05	42.62±10.40	——
CD4+/CD8+	1.53±0.75	1.38±0.58	1. 4 ~2. 0

Values presented as mean±SD, normal ranges cited from " Type 2 Diabetes Prevention Guide (2016 Edition, China)", "Guide of dyslipidemia prevention and treatment (2016 Edition) ", "Hypertension Prevention Guide (2016 Edition)" and "Establishment of reference range of peripheral blood T cell subsets in healthy people", "Percentage of Chinese people's lymphatic range" etc.

GLU: glucose; SSP: systolic pressure; DBP: diastolic pressure; TC: total cholesterol; TG: Triglyceride; HDL-C: High-density lipoprotein; LDL-C: Low-density lipoprotein; UA: Uric acid; BUN: Blood urea nitrogen; SCR: serum creatinine.

Figure S1. Composition of gut microbiota at the genus level

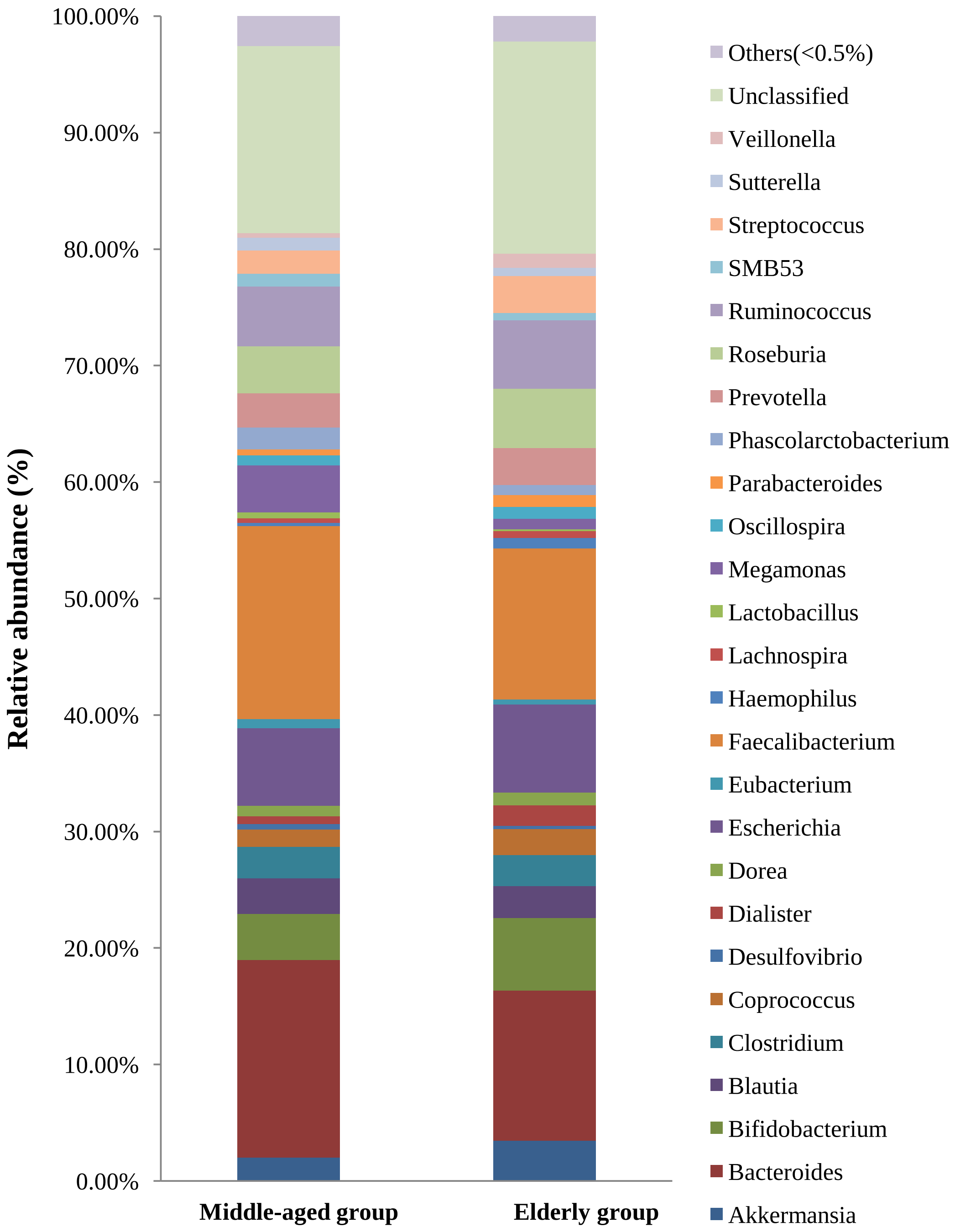

Genera with relative abundance greater than 0.5% are presented. Some unclassified genera are grouped as ‘Unclassified’. The lower abundant genera are grouped as ‘Others’.
